# The Unfolded Protein Response and Autophagy on the Crossroads of Coronaviruses Infections

**DOI:** 10.3389/fcimb.2021.668034

**Published:** 2021-04-28

**Authors:** Elisa B. Prestes, Julia C. P. Bruno, Leonardo H. Travassos, Leticia A. M. Carneiro

**Affiliations:** ^1^ Institut Necker Enfants Malades, Université Paris Descartes, Paris, France; ^2^ Laboratório de Inflamação e Imunidade, Instituto de Microbiologia Paulo de Goes, Universidade Federal do Rio de Janeiro, Rio de Janeiro, Brazil; ^3^ Laboratório de Imunoreceptores e Sinalização Celular, Instituto de Biofísica Carlos Chagas Filho, Universidade Federal do Rio de Janeiro, Rio de Janeiro, Brazil

**Keywords:** coronavirus, autophagy, unfolded protein response, integrated stress response, host-pathogen interaction

## Abstract

The ability to sense and adequately respond to variable environmental conditions is central for cellular and organismal homeostasis. Eukaryotic cells are equipped with highly conserved stress-response mechanisms that support cellular function when homeostasis is compromised, promoting survival. Two such mechanisms – the unfolded protein response (UPR) and autophagy – are involved in the cellular response to perturbations in the endoplasmic reticulum, in calcium homeostasis, in cellular energy or redox status. Each of them operates through conserved signaling pathways to promote cellular adaptations that include re-programming transcription of genes and translation of new proteins and degradation of cellular components. In addition to their specific functions, it is becoming increasingly clear that these pathways intersect in many ways in different contexts of cellular stress. Viral infections are a major cause of cellular stress as many cellular functions are coopted to support viral replication. Both UPR and autophagy are induced upon infection with many different viruses with varying outcomes – in some instances controlling infection while in others supporting viral replication and infection. The role of UPR and autophagy in response to coronavirus infection has been a matter of debate in the last decade. It has been suggested that CoV exploit components of autophagy machinery and UPR to generate double-membrane vesicles where it establishes its replicative niche and to control the balance between cell death and survival during infection. Even though the molecular mechanisms are not fully elucidated, it is clear that UPR and autophagy are intimately associated during CoV infections. The current SARS-CoV-2 pandemic has brought renewed interest to this topic as several drugs known to modulate autophagy – including chloroquine, niclosamide, valinomycin, and spermine – were proposed as therapeutic options. Their efficacy is still debatable, highlighting the need to better understand the molecular interactions between CoV, UPR and autophagy.

## Introduction

From single to multicellular, every living organism is frequently exposed to variable environmental conditions – including extremes of temperature, nutrient deprivation, irradiation, hypoxia, infections, and others – that can result in cell damage and/or dysfunction (Galluzzi et al., 2018). Eukaryotic cells have evolved mechanisms to cope with the stress generated by these conditions and support cellular functions, thereby maintaining microenvironmental and organismal homeostasis (Galluzzi et al., 2018). Such mechanisms include the DNA damage response (Jackson and Bartek, 2009), mitochondrial stress signaling (Chang et al., 2017), autophagy (Galluzzi et al., 2017) and the unfolded protein response (UPR), which is a component of the integrated stress response (Galluzzi et al., 2018) (ISR). The ISR is an evolutionarily conserved intracellular signaling network that can be initiated by several types of stress and converges to a common signaling hub – the phosphorylation of the α-subunit of the eukaryotic translation initiation factor 2, eIF2α (Costa-Mattioli and Walter, 2020). A family of four eIF2α-kinases is capable of sensing alterations in cellular homeostasis and respond by phosphorylating eIF2α: (i) double-stranded RNA (dsRNA)-dependent protein kinase (PKR) that is activated mainly by dsRNA during viral infection but also by oxidative and endoplasmic reticulum (ER) stress, growth factor deprivation, cytokines, bacterial infections, and ribotoxic stress (García et al., 2006); (ii) PKR-like ER protein kinase (PERK), which senses ER stress and also perturbations in calcium homeostasis, cellular energy or redox status (McQuiston and Diehl, 2017); (iii) heme-regulated eIF2α kinase (HRI), a sensor for low levels of intracellular heme as well as the formation of cytosolic protein aggregates, arsenite-induced oxidative stress, heat shock, nitric oxide, 26S proteasome inhibition, and osmotic stress (Girardin et al., 2020); and (iv) general control non-derepressible 2 (GCN2) that is activated in response to amino acid deprivation when it binds to deacylated transfer RNAs (tRNAs) *via* histidyl-tRNA synthetase-related domain (Battu et al., 2017). Not only do each of these pathways initiate crucial re-programming of the cell by modulating transcription of key genes and translation of new proteins, they also intersect with other stress-response pathways to restore homeostasis. Although ISR is primarily a homeostatic-preserving program by which cells adapt to survive, severe or long-lasting stress can induce cell death signaling by regulating autophagy or apoptosis.

The UPR is the cellular response to disturbances in the ER that ensues when its folding capacity is exceeded and unfolded proteins accumulate (Hetz, 2012). The ER is the primary site for the synthesis and folding of secreted and transmembrane proteins in eukaryotic cells. A myriad of environmental conditions (including nutrient deprivation, hypoxia, and loss of calcium homeostasis) can significantly alter the amount of proteins entering the ER, leading to UPR activation by one or more of three conserved ER-stress sensors: PERK, X-box-binding protein 1 (XBP-1) and activating transcriptional factor-6 (ATF-6) (Siqueira et al., 2018). These sensors display ER luminal domains capable of sensing modifications in the ER environment and cytosolic domains that trigger signaling pathways, resulting in reduced protein synthesis and increased ER folding capacity to ultimately preserve ER functions and cell viability. In addition, proteins that fail to correctly fold can be deployed to the ER distal secretory pathway, the ER-associated protein degradation (ERAD) pathway of the UPR (Hwang and Qi, 2018). In this case, misfolded proteins are retro-translocated from the ER back to the cytosol for degradation. Finally, in case of persistent stress, the UPR can initiate cell death programs.

Autophagy is another highly conserved process in which eukaryotic cells rely on to maintain cell homeostasis – in this case, by degrading and recycling cytoplasmic components, such as defective organelles or protein aggregates (Mizushima et al., 2008; Klionsky et al., 2014; Siqueira et al., 2018). The autophagosomes display a characteristic double-membrane structure that sequesters cytosolic targets, known as cargos, for degradation. Autophagy is carried out by at least 30 conserved ATG proteins divided into macromolecular complexes. Other hundreds of proteins have been shown to modulate this process in different contexts, including the master homeostatic regulator mechanistic target of rapamycin (mTOR). The formation of autophagosomes is divided into three successive stages: (i) initiation, which involves the ULK1-ATG13-FIP200 complex; (ii) membrane nucleation, that is dependent on the Beclin1(BECN1)-ATG14-PI3K complex, and (iii) membrane elongation that requires ATG8/LC3 lipidation. LC3 lipidation is a hallmark of autophagy and is established by a covalent linkage of cytosolic LC3 to the lipid phosphatidylethanolamine on the surface of the autophagosome, which enables autophagosome elongation and recruitment of autophagy targets (Reggiori and Klionsky, 2005; Carneiro and Travassos, 2013). The entire process, which has been named autophagic flux, is completed when autophagosomes fuse with lysosomes and the cargo is degraded in autophagolysosomes.

There are many parallels between ISR/UPR and mTOR/autophagy pathways, as they are both highly conserved signaling modules that regulate essential metabolic circuits, both in homeostatic and stress conditions. Also, the ER and autophagosomes are intrinsically linked since the former seems to be an essential source of lipids for the formation of the latter, and also provides a site for ATG14 anchoring, which plays a crucial role in recruiting the BECN1-ATG14-PI3K complex from the cytosol to sites of autophagosome initiation in response to starvation (Diao et al., 2015; Tan et al., 2016). Besides, localized phosphorylation of lipids by PI3K generates ER domains called omegasomes enriched in phosphatidylinositol-3-phosphate (Ptdlns3P) that recruit Ptdlns3P-effector proteins to generate sites for autophagosome nucleation and expansion (Mercer et al., 2018). In the context of ER stress, autophagy can be accessory by participating in the degradation of protein aggregates through ERAD (II), which is an alternative to the classic ubiquitin-proteasome ERAD, designated ERAD (I), that clears most soluble misfolded proteins (Rashid et al., 2015).

Both UPR and autophagy are often observed during viral infections, in some cases as a host cell defense mechanism limiting viral replication and, in others, contributing to viral replication and establishment of infection (Blázquez et al., 2014; Chan, 2014; Xiao and Cai, 2020). Viral infections are a major cause of cellular stress as viruses are able to manipulate several cellular processes to complete their replicative cycle (Ríos-Ocampo et al., 2018). In particular, the ability to divert the cell protein translation machinery to produce massive amounts of viral proteins profoundly impacts ER physiology (Gale et al., 2000). Furthermore, plus-stranded RNA viruses can pose an additional challenge for ER homeostasis as they synthesize their genome in association with extensive virus-induced rearrangements of intracellular membranes - including ER, endosomes or mitochondria (Romero-Brey and Bartenschlager, 2014). Included in this group are many viruses that cause disease in animals and humans, such as flaviviruses (for example, Dengue virus, Zika virus, Yellow fever virus, and West Nile virus), alphaviruses (for example, Chikungunya virus and Mayaro virus), and coronaviruses (for example, SARS-CoV, MERS-CoV, and SARS-CoV-2) (Jheng et al., 2014; Lee et al., 2018; Mehrbod et al., 2019). In the following sections of this review, we will discuss how UPR signaling and autophagy intersect in homeostatic conditions and in circumstances of mild and severe cell stress. In the final sections, we will take this discussion to the context of coronaviruses infections and highlight how these stress response pathways interfere with their replicative cycle.

## UPR and Autophagy in Homeostatic and Stress Conditions

Initially, PERK, IRE1, and ATF6 were considered to be active only when protein misfolding was detected in the cell. However, the identification of novel binding partners for these proteins suggest they participate in protein complexes that are important for regulating mitochondrial bioenergetics, cytoskeleton dynamics and membrane contacts (Hetz and Papa, 2018). For example, both PERK and IRE1 are present in mitochondria-associated membranes (MAMs) and the latter mediates the transfer of Ca^2+^ from the ER to mitochondria, thus regulating mitochondrial bioenergetics and physiology (MAMs) (Vliet et al., 2017; Urra et al., 2018; Carreras-Sureda et al., 2019). The absence of IRE1 in MAMs led to AMP-activated protein kinase (AMPK) phosphorylation and activation of autophagy, as determined by increased basal levels of LC3B, a key component of the autophagosomal membrane (Carreras-Sureda et al., 2019). PERK and IRE1 also directly interact with components of the actin cytoskeleton. PERK regulates intracellular Ca^2+^ fluxes by forming dimers upon detection of increased cytosolic Ca^2+^ levels and then interacting with filamin A to reorganize the cytoskeleton and contact sites between the ER and the plasma membrane (Vliet et al., 2017). Likewise, the dimerization of IRE1 promotes its interaction with filamin A and controls cell migration (Urra et al., 2018). Together, these recent studies show that, in addition to their role in UPR, dimerization of PERK and IRE1 also participate in cell homeostatic pathways unrelated to ER stress.

**Figure 1:**
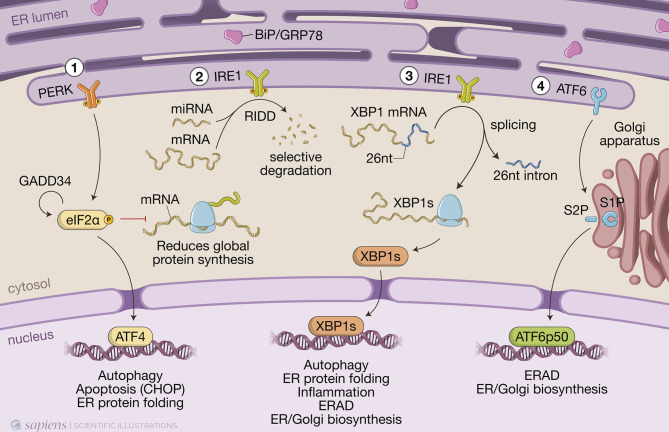
The three branches of the unfolded protein response (UPR). When misfolded proteins accumulate in the endoplasmic reticulum (ER) lumen, chaperone binding immunoglobulin protein (BiP/GRP78) is detached from the luminal domains of the three ER sensors to which it was bound, allowing PKR-like ER protein kinase (PERK) and inositol-requiring protein-1 (IRE1) to form homodimers and activating transcriptional factor-6 (ATF6) to transit to the Golgi. (1) PERK phosphorylates the α-subunit of the eukaryotic translation initiation factor 2 (eIF2α), resulting in a global reduction of protein synthesis while still maintaining translation of a few key proteins, such as activating transcription factor 4 (ATF4), which induces expression of genes involved in redox homeostasis, amino acid metabolism, protein synthesis, autophagy and apoptosis, such as the transcription factor C/EBP homologous protein (CHOP). Protein synthesis is restored when eIF2α is dephosphorylated by protein phosphatase 1 (PP1) regulatory subunit GADD34, which is also induced by ATF4 when ER stress is resolved. (2) IRE1 autophosphorylates to switch on its RNase activity, inducing a process known as regulated IRE1-dependent decay (RIDD), in which IRE1 cleaves and leads to the selective degradation of a small set of mRNAs or miRNAs. (3) IRE1 also excises a short 26-nucleotide intron from the mRNA encoding transcription factor X-box-binding protein 1 (XBP1), generating the spliced *Xbp1* mRNA, which is ultimately translated into the transcription factor XBP1s that, like ATF4, upregulates genes involved in multiple cell signaling pathways, such as ER-associated protein degradation (ERAD). (4) Full length ATF6 translocates from the ER to the Golgi, where it is cleaved by site-1 protease (S1P) and site-2 protease (S2P). This releases a cytosolic fragment which then transits to the nucleus, transcription factor ATF6p50, which drives a transcriptional program to reestablish homeostasis.

In conditions where misfolded proteins accumulate in the ER lumen, chaperone binding immunoglobulin protein (BiP/GRP78) is recruited and thus detached from the luminal domains of the three ER sensors to which it was bound, releasing PERK and IRE1 from their inactive monomeric states and allowing ATF6 to transit to the Golgi (Smith and Wilkinson, 2017) (**Figure 1**). Activation of the UPR triggers two distinct events to mitigate protein misfolding: a quick reaction that involves phosphorylation of targets to immediately reduce protein synthesis and increase protein degradation; and a more durable response consisting of transcriptional upregulation of hundreds of target genes to restore proteostasis (Hetz and Papa, 2018).

An instantaneous reaction to ER stress is initiated by dimerized PERK, which phosphorylates eIF2α in the cytosol leading to global attenuation of protein synthesis by forming an inhibitory complex with eIF2B that restricts its ability to bind to Met-tRNA initiator (Adomavicius et al., 2019). Paradoxically, at the same time, it initiates the translation of specific mRNAs that contain internal ribosomal entry sites. These include ATF4, which induces genes involved in redox homeostasis, amino acid metabolism, protein synthesis, apoptosis, and autophagy (Hetz et al., 2020). Under hypoxia, ATF4 promotes upregulation of LC3B through direct binding to a cyclic AMP response element-binding site in the *LC3B* promoter (Rzymski et al., 2010). This process likely has a protective role for the cell, as inhibition of autophagy, under those circumstances, led to metabolic consequences of hypoxic stress (Rzymski et al., 2010). ATF4 also induces the expression of CHOP and, together, they transcriptionally activate genes involved in autophagy. These include genes encoding proteins involved in the formation and maturation of the autophagosome, such as *Becn1* (which encodes BECN1) and genes from the ubiquitin-like protein (Ubl) system, like *Atg12* and *Map1-lc3b* (for LC3B) (B’chir et al., 2013). Genes encoding the activating enzyme (*Atg7*), the target of ATG12 attachment (*Atg5*), as well as genes encoding cargo receptors that are involved in specific degradation of ubiquitinated substrates, like *p62*, are also upregulated by the eIF2α/ATF4/CHOP branch (B’chir et al., 2013). Finally, protein synthesis is restored when eIF2α is dephosphorylated by protein phosphatase 1 (PP1) regulatory subunit GADD34, which is also induced by ATF4 when ER stress is resolved (Hetz et al., 2020).

ER stress also results in dimerization of the transmembrane protein kinase/endoribonuclease IRE1, which autophosphorylates to switch on its RNase activity that consists of excising a short 26-nucleotide intron from the mRNA encoding transcription factor XBP1 (Hetz et al., 2020). This processing generates the spliced *Xbp1* mRNA, which is ultimately translated into the transcription factor XBP1s that upregulates genes involved in ER protein translocation, folding and secretion, as well as degradation of misfolded proteins mainly by ERAD (Hetz et al., 2020). IRE1 also induces a process known as regulated IRE1-dependent decay (RIDD), in which IRE1 cleaves and leads to the degradation of a small set of mRNAs or miRNAs (Hetz et al., 2020) (**Figure 1**). IRE1 activates autophagy in a more indirect manner, by interacting with adapter proteins like tumor necrosis factor (TNF) receptor- associated factor 2 (TRAF2) and apoptosis signal-regulating kinase 1 (ASK1), forming the IRE1/TRAF2/ASK1 complex that activates c-Jun N-terminal kinase (JNK). JNK phosphorylates transcription factor c-Jun, which induces expression of *Becn1* (Senft and Ronai, 2015; Liu et al., 2020). Although activation of JNK by IRE1 promotes, at first, this protective autophagic pathway, during prolonged ER stress, it can turn into autophagy-dependent cell death (Liu et al., 2020; Lindner et al., 2020).

Upon sensing ER stress, full-length ATF6 (ATF6p90) translocates from the ER to the Golgi apparatus, where it is cleaved by site-1 protease (S1P) and site-2 protease (S2P) and releases a cytosolic fragment containing a basic leucine zipper (bZIP) transcription factor, ATF6p50, which then transits to the nucleus (Hetz et al., 2020) (**Figure 1**). ATF6p50 and XBP1s act simultaneously and may form heterodimers, driving specific gene expression programs that bring about chaperone activation and ER/Golgi biogenesis to increase the cell secretory capacity (Shoulders et al., 2013; Hetz et al., 2020).

As we have mentioned above, the type, intensity, and duration of circumstances that induce ER stress are critical in determining the cell fate upon UPR activation. Initially, the UPR attempts to resolve the misfolded protein build-up, but persistent and unresolved ER stress is bound to cause cell death by inducing apoptosis (Yao et al., 2020). For example, the different outcomes resulting from ATF4 activation make PERK and phosphorylated eIF2α crucial for determining the cell fate (Liu et al., 2020). Regarding the XBP-1 branch, while activation of *Xbp1* mRNA splicing is transient and attenuated after prolonged stimulation, the activity of RIDD can be sustained over time and eventually contribute to cell death (Hetz and Papa, 2018). It is important to note that although a variety of mechanisms by which UPR promotes apoptosis have been described, each UPR pathway contribution to this outcome is modest, suggesting the existence of cell-type specific networks that arbitrate the cell fate under severe ER stress. The mechanisms by which UPR regulates the balance between cell survival and apoptosis have been extensively reviewed elsewhere (Hetz and Papa, 2018; Hetz et al., 2020).

## UPR, Autophagy and CoV Infections

Coronaviruses (CoV) belong to the *Coronaviridae* family, which together with *Roniviridae* and *Arteviridae* form the order Nidovirales (Fung and Liu, 2019a). CoV infects an extensive range of birds and mammals, with several of them being economically important pathogens, including the avian infectious bronchitis virus (IBV) that causes severe respiratory and kidney diseases in poultry; the bovine coronavirus (BCoV) that causes respiratory tract diseases and diarrhea in cattle; feline infectious peritonitis virus (FIPV) that causes a fatal systemic disease in cats; and the transmissible gastroenteritis virus (TGEV) that causes diarrhea in pigs (Domańska-Blicharz et al., 2020). In humans, CoV are responsible for up to 30% of colds (Mesel-Lemoine et al., 2012). Importantly, CoVs have repeatedly demonstrated the ability to cross the species barrier and jump from non-human hosts to humans in a process known as zoonosis (Ye et al., 2020). In 2003, in the Chinese province of Guangdong, SARS-CoV-2 emerged as the etiological agent of the newly described severe acute respiratory syndrome (SARS), with high mortality rate. It was suggested that SARS-CoV was originated from bats and likely jumped to humans *via* some intermediate host (probably, palm civets) (Cheng et al., 2007). Nine years later, another zoonosis, this time originating from dromedaries, was detected in Saudi Arabia and named Middle-East respiratory syndrome (MERS), caused by MERS-CoV (Mohd et al., 2016). In neither case, the fear of a pandemic was confirmed. More recently, however, another SARS-inducing CoV - now named SARS-CoV-2 – emerged in Wuhan (China) to cause a pandemic that, to date, has infected 135 million people and killed more than 2,92 million people around the world (https://github.com/CSSEGISandData/COVID-19).

## CoV Replication: ER, Autophagy and the Origins of DMVs

CoVs are enveloped positive-sense RNA viruses (Chen et al., 2020). The first two-thirds of the genome consists of 2 large overlapping open reading frames, which encode 16 non-structural proteins (NSPs), including proteases, RNA-dependent RNA polymerase (prRdRp), RNA helicase, primase, and others, that form the viral replication and transcription complexes (RTCs), a platform to propagate viral mRNAs. The remaining portion of the genome includes interspersed open reading frames for the structural proteins – envelope (E), membrane (M), nucleocapsid (N), and the highly glycosylated spike (S) protein that projects from the viral envelope - as well as several accessory proteins generally nonessential for replication in tissue culture but capable of suppressing immune responses and enhancing pathogenesis (Ulferts et al., 2009). Infection begins when the viral S protein attaches to its complementary host receptor, angiotensin I converting enzyme 2 (ACE2) in the case of SARS-CoV-2, allowing the virus to enter the host cells by endocytosis or direct fusion of the viral envelope with the host membrane (Hoffmann et al., 2020). Once inside the cell, the virus induces massive rearrangement of the intracellular membrane network to generate double-membrane vesicles (DMVs) (Prentice et al., 2003). CoV then targets their RTCs on the DMV-limiting membranes through multi-spanning transmembrane proteins (NSP3, NSP4, and NSP6) (Reggiori et al., 2010). The sub-genomic viral RNAs are translated into structural and accessory proteins – transmembrane structural proteins (S, M, and E) are synthesized, inserted, and folded in the ER and transported to the ER-Golgi intermediate compartment (ERGIC), which is a structural and functional continuance of the ER, whereas N proteins are translated in the cytoplasm (Senger et al., 2020). Virion assembly occurs in the ERGIC, and particles are exported through a secretory pathway in smooth-wall vesicles, which ultimately fuse with the plasma membrane to release the mature virus particle (Fehr and Perlman, 2015).

Early on, a role for autophagy in RNA virus replication has been an attractive hypothesis because of its association with complex membrane rearrangements in the cytoplasm that can generate opposed double membranes. Indeed, DMVs resemble autophagosomes and are seen in large numbers in the cytosol of CoV-infected cells. In addition to DMVs, CoV replication complexes share other features of autophagosomes such as co-localization with multiple organelle markers and the acquisition of lysosomal markers throughout infection (Prentice et al., 2003). There have been different perspectives on the origin of CoV-induced DMVs – late endosomes, autophagosomes, and early secretory pathways have all been implicated as the membrane source of DMVs (Chen et al., 2020; V’kovski et al., 2020; Wolff et al., 2020). The major difficulty in solving this issue has been the lack or undetectable levels of marker proteins of subcellular organelles (Reggiori et al., 2011). Accumulating data now indicate that ER-derived membranes are the major source for DMVs formation: (i) several viral proteins, including proteins that are part of the RTCs such as NSP3 and NSP4, are glycosylated in the ER; (ii) also ectopically expressed NSP4 is found in the ER and moves to DMVs upon viral infection; (iii) blocking early steps of the secretory pathway abolishes CoV replication; (iv) electron tomography of cells infected with either SARS-CoV or mouse hepatitis virus (MHV) showed that DMVs are part of a reticulovesicular network of modified ER membranes with double-stranded RNA (dsRNA) inside; and, (v) the subunit of the ER translocon, Sec1α, is found on rearranged membranes during SARS-CoV infection (Knoops et al., 2008; Angelini et al., 2013). Finally, as we will discuss below, recent studies have suggested that DMVs biogenesis might be linked to ERAD tuning pathway. More recently, an effort to establish a compendium of host factors required for CoV infection, including SARS-CoV-2 and other seasonal CoV, identified an absolute requirement for the TMEM41B for infection with all the CoV tested. TMEM41B is a ER-transmembrane protein involved in autophagy, which, again, argues in favor of a role of the ER and components of the autophagy pathway in DMVs biogenesis (Schneider et al., 2021). Thus, CoV replication is structurally and functionally linked to the ER and autophagy.

## Autophagy: Is it Required or a Concomitant Event With CoV Replication?

Accumulated evidence has shown that CoVs interact differentially with components of the autophagic pathway with potential for both utilization of its components for replication and attenuation of autophagy but full understanding of their link to autophagy still awaits further investigation.

**Figure 2:**
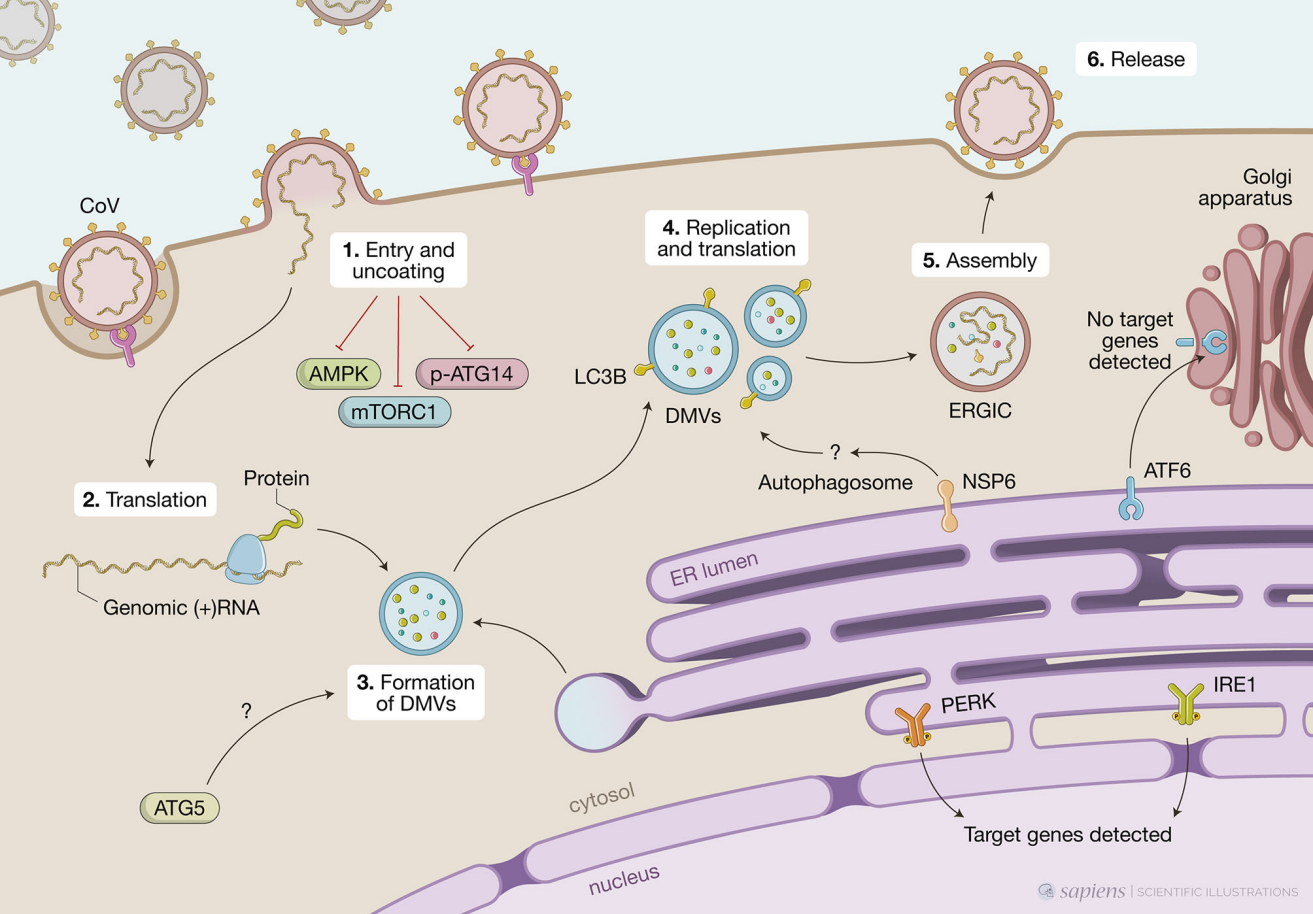
Unfolded protein response (UPR), autophagy and coronavirus (CoV) infections. This figure represents data obtained by using different cell models infected by CoV viruses mouse hepatites virus (MHV), infectious bronchitis virus (IBV), SARS-CoV or SARS-CoV-2. CoV infection begins when the viral S protein attaches to its complementary host receptor, allowing the virus to enter the host cells by endocytosis or direct fusion of the viral envelop with the host membrane. The process of SARS-CoV2 virus entry and uncoating (1) downregulates AMPK and reduces phosphorylation of ATG14, disrupting the autophagy flux, while simultaneously inhibiting mTORC1, which promotes autophagy. Once inside the cell, the viral positive-sense RNA is translated (2) and the virus induces massive rearrangement of the intracellular membrane network to generate double membrane vesicles (DMVs) (3). CoV-induced DMVs may originate from late endosomes, autophagosomes or vesicles from the early secretory pathway. MHV-induced autophagy was impaired in cells lacking ATG5, which displayed a deranged morphology of the membranes and decreased viral yield. Nonlipidated LC3 extensively colocalizes with DMV protein markers and downregulation of LC3 protects cells from CoV infection as result of defects in DMVs biogenesis. CoV replication occurs within DMVs and transmembrane structural proteins (S, M and E) are synthesized in the ER (4). Non-structural protein NSP6 from IBV, MHV and SARS-CoV are localized in the ER and participate in autophagosome formation *via* omegasomes, which could be used for DMV formation. Finally, new viral particles are transported to the ER-Golgi intermediate compartment (ERGIC) for assembly (5) and exported through secretory pathway in smooth-wall vesicles, which ultimately fuse with the plasma membrane to release the mature virus (6). A high demand for membranes for DMV formation and virion exocytosis contributes to ER stress. MHV activates PERK/eIF2α and IRE1 and target genes from these two branches are detected upon infection. Cleavage of ATF6 can also be observed but no target genes are detected subsequently.

MHV is considered a prototype CoV and has been extensively used to investigate mechanistic details of the replication and assembly of CoV *in vitro* and *in vivo.* Early studies have shown that MHV induced the formation of DMVs derived from autophagosomes and that proteins known to localize to replication complexes (p22 and N) colocalize with LC3^+^ foci in infected cells throughout the entire course of infection. MHV-induced autophagy was impaired in murine embryonic stem cells lacking ATG5, resulting in decreased viral yield (Prentice et al., 2003). In addition, *atg5* knockout cells displayed a deranged morphology of the membranes, particularly hyper-swollen RER containing multiple vesicles, but no autophagosome formation suggesting that the RER might be the source of membranes for replication complexes. The reconstitution of the *atg5* knockout with an expression plasmid restored viral yields implying the autophagy is required for viral replication (Prentice et al., 2003). Even though the molecular mechanisms were not determined, the authors hypothesized that the formation of DMVs could serve to sequester and concentrate viral replicases that were translated in the ER and that MHV may have evolved to utilize a preexisting cellular process – autophagy – to maximize replication efficiency. In contrast, Zhao et al. (2007) showed that in bone-marrow derived macrophages (BMMs) or primary mouse embryonic fibroblasts (MEFs) neither ATG5 nor an intact autophagic flux were required for MHV replication or release (Zhao et al., 2007). Considering viruses tropism to different cell types and the different permissiveness for viral replication among these cell types, it is possible that these discrepancies could be, at least in part, related to the different experimental models used (**Figure 2**).

Following studies suggested that CoV might not require an intact autophagic flux to replicate but still exploit components of the autophagic pathway to enhance infection. In this sense, another gene essential for autophagy, *atg7*, was shown to be dispensable for the formation of MHV-induced DMVs and viral replication in MEFs (Reggiori et al., 2010). Nevertheless, it was observed that endogenous nonlipidated LC3 extensively colocalized with the DMV protein markers, NSP2 and NSP3, and that downregulation of LC3, but not inactivation of host cell autophagy, protected cells from CoV infection as a result of defects in DMVs biogenesis. Of note, ectopically expressed GFP-LC3, which is widely used to track autophagosomes, colocalized with neither NSP2 nor NSP3 but ectopically expressed C-terminally HA-tagged, nonlipidable LC3 did (Reggiori et al., 2010). This observation distinguishes DMVs from autophagosomes as LC3 lipidation, which is indispensable for autophagosome elongation, is not required for its association with DMVs. This feature is reminiscent of ERAD tuning vesicles known as EDEMosomes. Post-translational regulation of ERAD factors contained in the ER lumen by rapid and selective removal, which is critical for ER homeostasis, is known as ERAD tuning. In addition to chaperones and folding enzymes, ER also contains ERAD factors that recognize non-native proteins, extract them from the folding machinery and ensure their transport for proteasomal degradation. The ERAD regulators EDEM1, OS-9, and others are removed from the ER in vesicles that display LC3-I in their limiting membrane – the EDEMosomes - and degraded by endo-lysosomal enzymes. Based on this, the authors propose a mechanism by which MHV hijacks the ERAD tuning pathway to coopt cellular membranes for DMV formation and support viral RTCs. Indeed, MHV infection caused accumulation of EDEM1 and OS-9 in the DMVs, and this was independent of autophagy as *atg7* deletion did not affect the intracellular levels of EDEM1 as it did those of p62, an autophagy substrate. Importantly, viral-coopting of this cellular process blocks the normal clearance of these vesicles as illustrated by data showing that, in non-infected cells, EDEM1 has a half-life of about 1 hour but upon MHV infection, is still found after several hours of infection even with virus-induced host translational shutoff, indicating that there is actually defective clearance of EDEM1 in infected cells.

A screen using individual IBV non-structural proteins for their ability to induce autophagy showed that NSP6 was located to the ER and induced ER puncta containing DFCP1 and ATG5 (Cottam et al., 2011). NSP-6-induced autophagosomes required ATG5 and the recruitment of lipidated LC3-II, features of classic autophagosomes generated in the ER *via* omegasomes rather than EDEMosomes. Furthermore, class 3 PI3 kinase activity was also required, indicating PtdIns(3)P are generated from ER lipids to build phagophores. In the IBV model, the infection or ectopic expression of NSP6 induces a complete autophagic flux as the autophagosomes are fused with Lamp1-positive vesicles, which show their ability to deliver the cargo to the lysosomes. However, an intact autophagy flux is not required for viral replication as this was unaffected by pharmacological autophagy inhibitors or silencing of ATG5. NSP6 orthologs from other CoV (SARS-CoV and MHV) also localized to the ER, from where they generate autophagosomes *via* an omegasome intermediate. Targeting NSP6 to the ER resulted in partial XBP1 splicing and undetectable increase in CHOP expression, indicating that ER stress is limited and not mechanistically involved in autophagy induction (Cottam et al., 2014). It would be interesting to investigate other branches of the UPR since, as we mentioned above, individual pathways might not represent the whole picture.

The same group investigated the characteristics of the autophagosomes induced by ectopic expression of NSP6 from several CoV (SARS-CoV, MHV or IBV) as well as IBV infection (Cottam et al., 2014). All of them induced the formation of a significant number of autophagosomes that were significantly smaller (< 0.5 μm diameter) than the ones induced by starvation (> 1.0 μm). Even with concomitant signals for autophagy activation, such as starvation or mTOR inhibition, IBV infection or IBV NSP6 ectopic expression limited expansion of omegasomes, thereby limiting the expansion of autophagosomes. The authors showed that mTOR was missing from the surface of lysosomes of cells expressing NSP6 even when cultured in nutrient media and suggested that NSP6 prevents mTOR association with lysosomes limiting the formation of large autophagosolysosomes. It still to be addressed if NSP6 acts by directly interacting with other proteins at the surface of lysosomes or indirectly by affecting other signaling pathways. Although smaller, NSP6-induced autophagosomes could still take up SQSTM1/p62 and deliver it to lysosomes. Additional work will be necessary to understand if limiting autophagosome expansion brings any advantage for CoV replication. Given that the size of DMVs is much smaller than what is observed in starvation-induced autophagosomes, the authors hypothesize that these smaller autophagosomes could be used to generate DMVs (Cottam et al., 2014).

In contrast to the studies above that suggest a positive correlation between the induction of autophagy and CoV replication, other studies have proposed an inhibitory effect of CoV on the autophagic process. For instance, MERS-CoV seems to establish a tug of war in which at the same time that autophagy limits viral propagation, the virus is able to impair autophagic flux by activation the E3-ligase S-phase kinase-associates protein 2 (SKP2) (Gassen et al., 2019). SPK2 acts *via* FKBP5, a stress-regulated protein involved in numerous pathways through scaffolding regulatory protein interactions and the involvement of AKT1 and PHLPP. Activated phosphorylated SPK2 polyubiquitinates the critical autophagy initiating protein BECN1 for proteasomal degradation. Conversely, pharmacological inhibition of SPK2 stabilizes BECN1, enhances autophagy, and restricts MERS-CoV propagation. Altogether these results indicate that autophagy is a relevant anti-MERS-CoV mechanism. Indeed, knocking down ATG5 resulted in a 52-fold increase in the formation of MERS-CoV particles (Gassen et al., 2019). More recently, the same authors have shown that similar to MERS-CoV, SARS-CoV-2 strongly reduced the autophagic flux in two different cell lines – the human bronchial epithelial cells NCI-H1299 and Vero cells (Gassen et al., 2020). This later study showed that the phosphorylated active forms of AMPK, AMPK substrates (LXRXX), AMPH downstream targets (TSC2 and ULK1) and mTORC1 were all downregulated upon SARS-CoV-2 infection. Concomitantly, increased levels of phosphorylated AKT1 were observed, leading to SKP2 activation and decreased BECN1 levels. This resulted in reduced ATG14 phosphorylation and oligomerization and subsequent lack of fusion of autophagosomes and lysosomes and disrupted autophagy flux. It had been previously suggested that the membrane-associated papain-like protease PLP2 (PLP2-TM) of SARS-CoV or MERS-CoV could be involved in blockage of autophagosomes-lysosomes fusion and suppression of the autophagic flux (Yang and Shen, 2020).

## ER Stress Induces Different Signaling Circuits in Response to CoV Infection –

Despite the lack of consensus on the biogenesis of DMVs, it is clear that its membranes originate from the ER, whether it is through omegasomes/autophagosomes, EDEMosomes or both. Such high demand for membranes represents an additional burden to the ER that is also coopted to produce viral proteins. Indeed, during CoV replication, massive amounts of structural proteins are synthesized in the ER, and the production, folding and modification (in particular, extensive glycosylation of S protein) of these proteins increase the workload of the ER, eventually overloading its folding capacities and leading to UPR activation. Continuous depletion of the ER lipid content due to virion exocytosis also contributes to ER stress. Several pieces of evidence indicate that CoV infection induces ER stress: (i) genes and proteins involved in ER stress were shown to be upregulated in cells infected with SARS-CoV. Different studies showed that both the glucose-regulated protein 94 (GRP94) and GRP78/BiP are induced upon SARS-CoV infection in cell culture systems (Chan et al., 2006; Palmeira et al., 2020). In addition, infection with either MHV or SARS-CoV also results in up-regulation of homocysteine-inducible, ER-stress inducible, ubiquitin-like domain member 1 (HERPUD1), an ER stress marker (Fung and Liu, 2014).

Among the three branches of UPR, the PERK-eIF2α is the best characterized in CoV-infected cells. For example, MHV induces significant eIF2α phosphorylation and ATF4 upregulation in infected cells resulting in sustained translation repression. Even though there have been conflicting results in early studies on the role of the integrated stress response on CoV infections, it appears that both PERK and PKR, and subsequently eIF2α phosphorylation, occur at the early stages of CoV infections in cell cultures. In cells infected with SARS-CoV, PERK, PKR and eIF2α phosphorylation were detected as early as 8h post-infection (Krähling et al., 2009). Even though it significantly inhibited SARS-CoV-induced apoptosis, the knock-down of PKR did not affect eIF2α suggesting that this could be dependent on PERK activation. Supporting this is the previous observation that SARS-CoV accessory protein 3a induces PERK phosphorylation (Minakshi et al., 2009). Two complementary studies from the same group indicated that the PERK/PKR-eIF2α pathway is also activated at early stages of IBV infection *in vitro*, leading to ERK1/2 phosphorylation and promoting cell survival. After 8 h of infection, however, eIF2α is dephosphorylated as a feedback response due to the accumulation of GADD34, which is downstream of ATF4 and GADD153. Up-regulation of GADD153, which was partially blocked by silencing either PKR or PERK, also promoted apoptosis in IBV-infected cells (Wang et al., 2009; Liao et al., 2013). Altogether, these results highlight the delicate balance between cell survival and cell death upon UPR activation depending on the intensity and/or duration of stress.

The ORF3 protein of porcine epidemic diarrhea virus (PEDV), similar to SARS-CoV 3a protein or human pathogenic coronavirus NL63 (hCoV-NL63) ORF protein, localizes to the ER and triggers ER stress by upregulating GRP78 and activating the PERK-eIF2α pathway (Zou et al., 2019). This, in turn, results in induction of autophagy as shown by conversion of LC3-I into LC3-II. ORF3 protein is thought to function as an ion channel and to influence virus production and virulence (Wang et al., 2012).

Studies using MHV have demonstrated that the IRE1 axis also senses ER stress during infection, as shown by efficient splicing of XBP1 mRNA upon infection or overexpression of the S protein (Bechill et al., 2008). However, how this contributed to the cellular response was unclear since the protein product of the spliced XBP1 was not observed and genes known to be downstream of XBP1s – such as EDEM1, ER DNA J domain-containing protein 4 (ERdj4), and protein kinase inhibitor of 58 kDa (p58^IPK^) – were not significantly up regulated following infection. It is possible that sustained eIF2α phosphorylation and translational repression observed during MHV infection interfere with the translation of the XBP1 protein. Another possibility is that IRE1 operates through alternative signaling pathways. It has been demonstrated that activation of IRE1 was essential for autophagy induction upon infection with another CoV, IBV (Fung and Liu, 2019b). Autophagosome formation and autophagic flux were also dependent on ATG5 but independent of BECN1. In this model, XBP1 splicing was dispensable, and IRE1 signaled through ERK1/2 to modulate autophagy induction in infected cells and protect them from apoptosis. A more recent study demonstrated that IBV can induce significant splicing of XBP1 mRNA and subsequent upregulation of EDEM1, ERdj4 and p58^IPK^ in various cell lines (Fung et al., 2014). This was dependent on IRE1 activation as inhibiting or knocking down IRE1 effectively blocked IBV-induced XBP1 mRNA splicing and effector genes upregulation. In this context, the IRE1-XBP1 pathway seems to protect cells from apoptosis by modulating JNK and AKT phosphorylation. Altogether, these studies highlight how stress-related pathways intersect in different ways depending on cellular context and how different signaling circuits ultimately define cell fate. Also, it is important to evaluate the cell response as a whole, as it is not uncommon for RNA viruses to utilize only a subset of the components of stress response pathways that can signal through alternative pathways.

Similar to what was observed with IRE1, significant cleavage of ATF6 is observed following infection with MHV but the activation of target genes was not detected using luciferase reporter constructs under the control of ERSE promoters (Fung et al., 2014). In addition, ATF6 levels (full length and cleaved) significantly decrease at later time points of infection. The authors of this study suggest that global protein synthesis arrest following eIF2α phosphorylation impedes ATF6 accumulation and subsequent activation of target genes (Bechill et al., 2008).

ATF6 was implicated in the response to SARS-CoV. On the 2003 SARS-CoV outbreak, the accessory protein 8ab was found in animals and early human isolates. This protein was found to co-immunoprecipitate with and induce ATF6 cleavage and nuclear translocation (Sung et al., 2009). At the peak of the epidemic, human isolates presented a 29-nt deletion in the middle of ORF8, resulting in two smaller ORFs that encode the truncated polypeptides ORF8a and ORF8b. More recently, it was demonstrated that ORF8b forms insoluble intracellular aggregates leading to ER stress, lysosomal damage, and subsequent activation of the master regulator of the autophagy and lysosome machinery, transcription factor EB (TFEB), leading to increased autophagic flux. ORF8b aggregates are partially degraded by the autophagy-lysosome pathway. Depending on the cell type this may result in either cell death (as observed in epithelial cells) or a robust NLRP3 inflammasome activation by directly targeting its LRR domain with the release of inflammatory mediators (as observed in macrophages). In wild-type macrophages, ORF8b induces NLRP3-dependent pyroptosis while in NLRP3-deficient macrophages, cell death results from mitochondrial dysfunction (Shi et al., 2019).

## Manipulation of UPR and Autophagy as a Strategy to Limit CoV Replication

As the current SARS-CoV-2 pandemic progressed, the repurposing of drugs already approved for human use could represent the fastest way to limit viral spread and/or severe disease, save lives, and prevent the collapse of health care systems. Drugs known to modulate both UPR (e.g. thapsigargin) (Al-Beltagi et al., 2021) and autophagy (e.g. hydroxichloroquine) (Senger et al., 2020) were shown to be broad-spectrum inhibitors for several respiratory viruses, including CoV, and emerged as candidates. Even though experimental findings have not yet been translated into efficient treatment and the use of these drugs remains controversial, the literature offers a rationale to target UPR and autophagy in the context of CoV infections. As we have described in this review, these two events are an integral part of the host cell-virus interactions and can be involved in different steps of the viral replicative cycle – from the establishment of replicative niches (DMVs) to regulating cell death. In particular, viral usage of double membranes vesicles resembling autophagosomes as a platform for replication, as a source of membrane for their envelope, as well as an intracellular shuttle for their exocytosis has been reported. Even though the precise mechanisms of DMVs biogenesis are still a matter of debate, components of the autophagic machinery and/or autophagic flux are often found to be necessary. Finally, all CoV identified so far have evolved to manipulate the autophagy pathway in some way, which once again argues that this pathway is essential for viral replication cycle.

One of the first proposed clinical trials in the wake of the COVID-19 pandemic draw huge worldwide attention to the putative benefits of hydroxychloroquine (HCQ) in the early treatment of patients infected with SARS-CoV-2 (Senger et al., 2020). Previously, HCQ had been shown to inhibit MERS-CoV replication *in vitro* in a screening of FDA-approved compound library (Touret et al., 2020). In experimental SARS-CoV infections, HCQ was able to limit inflammation markers even though it has not affected viral titers in the lungs of infected mice (Maisonnasse et al., 2020). The mechanisms proposed to explain the effects of HCQ on SARS-CoV-2 infection included the ability to interfere with ACE2 terminal glycosylation by raising the pH in the Golgi compartment thereby reducing the cell surface expression of SARS-CoV2 receptor (Kalra et al., 2020). In addition, by accumulating in the acidic organelles such as endosomes and lysosomes and neutralizing their pH, it can also disrupt fusion of viral endosomes with lysosomes or the activity of proteases preventing the cleavage of S protein blocking early steps in the viral life cycle (Chen and Geiger, 2020). Finally, HCQ is a well-known inhibitor of autophagy flux by impairing autophogasome fusion with lysosomes (Mauthe et al., 2018). After months of intense debate worldwide and follow-up studies, the efficacy of HCQ to treat COVID-19 was not confirmed, and its use has been discouraged by the FDA because of the risk of side effects. Nevertheless, one should not disregard autophagy as a potential pharmaceutical target because (i) it is a critical host process that controls all steps harnessed by SARS-CoV-2 and (ii) a collection of other drugs known to be autophagy modulators were shown to reduce or block SARS-CoV-2 infection *in vitro.* For instance, work from Gorshkov et al. (2020) identified 6 compounds known to be autophagy modulators that were able to reduce the cytopathic effects of SARS-CoV-2 *in vitro* with EC_50_ values ranging from 2.0 to 13 μM and selectivity indices ranging from 1.5 to >10-fold and their efficacy for inhibiting autophagy correlated with their ability to prevent SARS-CoV-2 cytopathic effects in various cell lines (Gorshkov et al., 2020).

As mentioned previously in this review, work from Gassen et al. (2019) showed that in their experimental conditions, MERS-CoV and SARS-CoV-2 impair autophagy by targeting BCL-1 for proteasomal degradation upon activation of SKP2. Blocking SKP2 with different compounds, including FDA-approved drugs, stabilized BECN1 limiting MERS-CoV and SARS-CoV-2 propagation which, in this case, contrary to HCQ that inhibits autophagy, indicates that boosting autophagy could be an efficient anti-CoV mechanism (Gassen et al., 2019). These authors tested a collection of known autophagy modulators such as spermidine, spermine, rapamycin, AKT1 inhibitors such as MK-2206, and the BCN1 stabilizing antihelmintic drug niclosamide and further advanced the idea that inducing autophagy can limit SARS-CoV infections (Gassen et al., 2020).

Manipulating the UPR has also been shown to be a potential strategy to treat infections with respiratory viruses. Al-Beltagi et al. showed that, *in vitro*, non-cytotoxic levels of thapsigargin (TG), an inhibitor of the ER-Ca ATPase pump, can block the replication of relevant human respiratory viruses such as CoV (including SARS-CoV-2), Respiratory Syncytial Virus and Influenza (Al-Beltagi et al., 2021). The effects of TG on CoV (hCoV-229E, MERS-CoV and SARS-CoV-2) replication have also been reported in another recent study (Shaban et al., 2020). The authors had previously shown that, in the case of CoV OC43 and RSV, TG-induced ER stress leading to UPR was a central innate immune driver that mediated several downstream host antiviral mechanisms that are particularly effective in blocking the replication of different RNA viruses, which include increased expression of ER stress genes (*DDIT3, HSPA5* and *HSP90B1*) and was accompanied by reduced viral transcription and viral protein expression (Goulding et al., 2020).

## Concluding Remarks

As we understand cellular stress responses, it becomes clear that multiple pathways that are activated independently intersect in different ways, resulting in specific signaling circuits tailored to the cellular context. In this sense, it has been recently shown that there is a crosstalk between UPR and autophagy even in homeostatic conditions and it can be differentially modulated upon mild and severe or long-lasting stress. In the context of infections, stress-response mechanisms play multiple roles, including maintenance of homeostasis, fine-tuning of the immune response, and, in some cases, a direct anti-infectious role. The UPR/ISR and autophagy are known to limit the replication of several viruses. On the other hand, many RNA viruses, including CoV, take advantage of these responses to enhance their replication. There has been much debate on the biogenesis of CoV DMVs, which is a crucial step of the viral replicative cycle. Despite the lack of consensus, it is clear that its membranes originate from the ER, whether through omegasomes/autophagosomes, EDEMosomes or both. In addition, even though a complete autophagic flux seems to be dispensable, components of the autophagic machinery may be required in “alternative” pathways for CoV DMVs formation and replication. Similarly, ER stress induced upon CoV infection signals both through canonical UPR response as well as “alternative” signaling involving MAP kinases. There have been enough pieces added to the puzzle to establish a role for UPR and autophagy in CoV replication. However, the missing pieces will define the signaling circuits involved. This will be particularly important for the design of vaccines and therapeutic strategies to face the pandemic.

## Author Contributions

EP, JB, LT, and LC conceived and wrote the manuscript. LC revised the manuscript. All authors contributed to the article and approved the submitted version.

## Funding

EP is supported by a fellowship from Coordenação de Aperfeiçoamento de Pessoal de Nivel Superior (CAPES). JB is supported by a studentship from Fundação Carlos Chagas Filho de Amparo à Pesquisa do Estado do Rio de Janeiro (FAPERJ). Research in the labs of LT and LC are funded by Conselho Nacional de Desenvolvimento Científico e Tecnológico (CNPq) and FAPERJ.

## Conflict of Interest

The authors declare that the research was conducted in the absence of any commercial or financial relationships that could be construed as a potential conflict of interest.
